# Mucinous Tubular and Spindle Cell Carcinoma of the Kidney: A Study of Clinical, Imaging Features and Treatment Outcomes

**DOI:** 10.3389/fonc.2022.865263

**Published:** 2022-04-11

**Authors:** Xiaofeng Xu, Jing Zhong, Xiumin Zhou, Zhifeng Wei, Qiuyuan Xia, Pengfei Huang, Changjie Shi, Jianping Da, Chaopeng Tang, Wen Cheng, Jingping Ge

**Affiliations:** ^1^ Department of Urology, Jinling Hospital, School of Medicine, Nanjing University, Nanjing, China; ^2^ Department of Radiology, Jinling Hospital, School of Medicine, Nanjing University, Nanjing, China; ^3^ Department of Oncology, The First Affiliated Hospital of Soochow University, Suzhou, China; ^4^ Department of Urology, The Fourth Affiliated Hospital of Nanjing Medical University, Nanjing, China; ^5^ Department of Pathology, Jinling Hospital, School of Medicine, Nanjing University, Nanjing, China; ^6^ Department of Ultrasound Diagnosis, Jinling Hospital, School of Medicine, Nanjing University, Nanjing, China

**Keywords:** mucinous tubular and spindle cell carcinoma, renal cell carcinoma, kidney, imaging features, prognosis

## Abstract

**Purpose:**

To describe the clinical, imaging, pathological features and oncologic outcomes of mucinous tubular and spindle cell carcinoma (MTSCC) of the kidney.

**Patients and Methods:**

Twenty-two cases of MTSCC were pathologically identified between January 2004 and April 2021 at our institution. The clinical and imaging findings, pathological features, treatment methods and outcomes of the patients were reviewed.

**Results:**

These cases included 17 women and 5 men, with a median age at diagnosis of 52.5 years. On contrast-enhanced CT, MTSCC was less enhanced than the adjacent renal parenchyma. Tumor attenuation values were 33.3 ± 6.8HU, 44.0 ± 9.1HU, 54.4 ± 13.9HU and 67.1 ± 11.8HU in the non-contrast, corticomedullary, nephrographic and excretory phases of CT, respectively. Contrast-enhanced ultrasonography and MRI also showed hypovascular features of the masses. On MRI, the tumors were isointense on T1-weighted images and slightly hypo- or hyperintense on T2-weighted images. Diffusion-weighted imaging revealed a low apparent diffusion coefficient of the tumor. The patients were managed with laparoscopic partial nephrectomy (n=5), radical nephrectomy (n=16), or robotic-assisted laparoscopic partial nephrectomy (n=1). The median follow-up time was 59.5 months. All the patients were free of local recurrence or distant metastasis.

**Conclusions:**

MTSCC is generally indolent and has favorable outcomes. The imaging features of MTSCC are generally hypovascular, which is significantly different from clear cell renal cell carcinoma. However, it is still difficult to distinguish MTSCC from other hypovascular renal tumors preoperatively because their imaging features overlap. Further studies are essential to fully characterize the features of this rare RCC variant.

## Introduction

Mucinous tubular and spindle cell carcinoma (MTSCC) is a rare subtype of renal cell carcinoma (RCC). It was recognized as a distinct RCC entity in the 2004 World Health Organization Classification of Renal Tumors (1). Owing to the rarity of this renal tumor, referential researches are limited. The clinical and imaging features and prognosis of MTSCC have not been clearly described. We retrospectively reviewed the clinical data of the patients diagnosed with MTSCC at our institution between January 2004 and April 2021. This study aimed to characterize the clinical, imaging, pathological features, and prognosis of MTSCC.

## Patients and Methods

Approved by the Institutional Review Board of Jinling Hospital, the database of our institution was queried retrospectively. And the requirement for informed consent was waived. Twenty-four patients were initially identified from pathological reports. Two patients were subsequently excluded after further pathological re-review. The final dataset included 22 cases identified as MTSCC. Demographic data, relevant imaging data, treatment received, and pathological and prognostic data were collected.

Fifty imaging studies were available for review: 19 ultrasound, 12 unenhanced CT scans, 15 contrast-enhanced CT scans, 2 unenhanced MRI scans and 2 contrast-enhanced MRI scans. Preoperative CT and MRI scans were evaluated using a picture archiving and communication system (PACS) workstation. A small circular region with the most obvious enhancement of the tumor in the corticomedullary phase was selected as the region of interest (ROI) on CT. Areas of necrosis or calcification were avoided. The same ROI was analyzed for all sequences. A suitable ROI on the renal parenchyma was selected and remained consistent throughout all phases. Hounsfield units (HU) of ROI were measured. Tumor was defined as exophytic if more than 50% of it was outside of the expected normal contour of the kidney, partially exophytic if <50% of it was outside, and endophytic if the mass was completely within the renal contour.

The operative information was reviewed. The tumors were staged according to the 2017 American Joint Committee on Cancer (AJCC) tumor node and metastasis (TNM) classification (2). Other pathological parameters including tumor size, necrosis, hemorrhage and sarcomatoid changes were also noted. The duration of follow-up was calculated from the date of diagnosis to the date of the last follow-up or death.

Data analysis was performed using the Statistical Package for the Social Sciences (SPSS), version 26 (SPSS Inc.; IBM Corp., Armonk, NY, USA). The diameters of the tumors with different homogeneity of enhancement on CT are presented as mean ± SD and were compared using Student’s t-test as applicable. Statistical significance was set at p values < 0.05.

## Results

### Patient Characteristics and Clinical Features

The baseline characteristics of the patients are summarized in **Table 1**. There was a preference for women. Seventeen patients (77.3%) were female. The median age of the patients at diagnosis was 52.5 years (range 32-66). The median tumor size was 4.3 cm (range 1.9-11.4). Twenty patients (90.9%) had asymptomatic incidentally discovered tumors. Two patients (9.1%) presented with local symptom of flank pain. None of the patients had bilateral masses.

**Table 1:** Patient characteristics and clinical presentation.

Variable	Value
Total no. of cases	22
Female, n(%)	17 (77.3%)
Age at diagnosis, years, median(range)	52.5 (32-66)
Left-sided tumor, n(%)	11 (50%)
Tumor size, cm, median(range)	4.3 (1.9-11.4)
BMI, kg/m^2^, median(IQR)	24.3 (21.7-25.4)
Symptoms at presentation	
None, n(%)	20 (90.9%)
Localized, n(%)	2 (9.1%)
Systemic, n(%)	0 (0.0%)
Metastases at presentation, n(%)	0 (0.0%)
Preoperative hemoglobin, g/L, median(IQR)	127 (119.5-134.8)
Hemoglobin below lower limit of normal, n(%)	6 (27.3%)
Preoperative LDH, IU/L, median(IQR)	163 (151.8-178)
LDH above upper limit of normal, n(%)	0 (0.0%)

IQR, interquartile range; BMI, body mass index; LDH, lactate dehydrogenase.

### Ultrasonography Features

Eight patients underwent ultrasound examination, including conventional abdominal ultrasound, color Doppler ultrasound and contrast-enhanced ultrasonography (CEUS). Four patients underwent conventional abdominal ultrasonography and color Doppler ultrasonography. One patient underwent conventional abdominal ultrasonography and CEUS. Six patients underwent conventional ultrasonography only (**Table 2**).

**Table 2:** US characteristics of primary tumor.

Case No.	Echogenicity	Margin	CDFI	CEUS
1	Homogenously hypoechoic	Well-marginated	–	–
2	Homogenously hypoechoic	Well-marginated	–	Slow in and fast out, heterogeneous less
3	Homogenously hypoechoic	Well-marginated	A small amount of peripheral blood flow	Slow in and simultaneous out, heterogeneous less
4	Heterogeneously hypo and anechoic	Well-marginated	–	Slow in and simultaneous out, heterogeneous less
5	Homogenously hypoechoic	Well-marginated	No blood flow	Slow in and fast out, heterogeneous less
6	Homogenously hypoechoic	Well-marginated	A small amount of peripheral blood flow	Simultaneous in and fast out, homogeneous less
7	Homogenously hypoechoic	Well-marginated	A small amount of peripheral blood flow	Slow in and fast out, heterogeneous less
8	Homogenously hypoechoic	Well-marginated	No blood flow	–
9	Homogenously hypoechoic	Well-marginated	–	–
10	Homogenously hypoechoic	Well-marginated	–	–
11	Homogenously hypoechoic	Well-marginated	A small amount of peripheral blood flow	–
12	Homogenously hypoechoic	Well-marginated	–	–
13	Homogenously hypoechoic	Well-marginated	–	–
14	Homogenously hypoechoic	Well-marginated	No blood flow	Slow in and fast out, heterogeneous less
15	–	–	–	–
16	Homogenously hypoechoic	Well-marginated	–	–
17	Heterogeneously hypo and hyper echoic	Well-marginated	No blood flow	Slow in and simultaneous out, heterogeneous less
18	Homogenously hypoechoic	Well-marginated	A small amout of peripheral blood flow	–
19	–	–	–	–
20	Homogenously hypoechoic	Well-marginated	No blood flow	–
21	Homogenously hypoechoic	Well-marginated	A small amount of peripheral blood flow	Slow in and fast out, heterogeneous less
22	–	–	–	–

CDFI, color Doppler flow imaging; CEUS, contrast-enhanced ultrasonography.

Conventional abdominal ultrasound showed that all masses appeared to be well-marginated. Most of the tumors (17/19) were homogeneously hypoechoic. The other two heterogeneous masses were mixed hypoechoic and anechoic. Color Doppler ultrasound images revealed no obvious blood flow within the masses. There was only a small amount of peripheral blood flow signal around the masses. CEUS showed that all tumors were less enhanced than the adjacent renal parenchyma. And most of their enhancement (8/9) were heterogeneous and later than that of the adjacent renal cortex. Only one tumor was homogeneously and simultaneously enhanced with the adjacent renal parenchyma. The washout of the tumors was earlier (6/9) or simultaneous (3/9) than that of the adjacent renal parenchyma.

### Radiological Findings

Twelve patients underwent CT in all four phases: non-contrast (NC), corticomedullary (CM), nephrographic (Ne) and excretory (Ex) (**Table 3**). Other three patients were scanned in CM, Ne and Ex phases. All tumors grew expansively with a spherical or ovoid shape on CT images. All masses had well-demarcated margins. Six of the 15 tumors showed lobulated contours. The growth patterns of the tumors were exophytic (n=5), partially exophytic (n=8) and completely endophytic (n=2). Four tumors contained cystic and necrotic components. Four tumors contained calcification. The mean attenuation values of the tumor were 33.3 HU, 44 HU, 54.4 HU, 67.1 HU in NC, CM, Ne and Ex phases of CT scan, respectively. The mean attenuation values of the normal renal cortex were 36.2HU, 138.1HU, 155.3HU and 129.5 HU in NC, CM, Ne and Ex phases, respectively (**Table 4**). Nine of 15 masses were homogeneously enhanced and had a mean diameter of 4.7 ± 1.9cm (range 2.6-8.3). And the mean diameter of the tumors enhanced heterogenously (6/15) was 6.9 ± 3.6cm (range 3.0-11.4). However, there was no statistically significant difference between the two groups (P=0.21). Enhancement of all masses was mild, slow and progressive.

**Table 3:** CT characteristics of primary tumor.

Case No.	Side	Size (cm)	CTD (HU)												Enhancement homogeneity
			NC.t	CM.t	Ne.t	Ex.t	NC.c	CM.c	Ne.c	Ex.c	NC.m	CM.m	Ne.m	Ex.m	
1	R	4.0	17	20	22	42	37	75	82	122	21	44	39	121	Heterogenous
2	R	3.0	–	–	–	–	–	–	–	–	–	–	–	–	–
3	L	3.8	–	–	–	–	–	–	–	–	–	–	–	–	–
4	L	1.9	–	–	–	–	–	–	–	–	–	–	–	–	–
5	R	2.6	28	42	54	68	33	126	133	113	28	41	58	135	Homogenous
6	L	6.3	36	40	46	54	36	136	123	108	29	45	66	138	Homogenous
7	R	9.0	–	39	42	63	–	142	141	116	–	65	92	153	Heterogenous
8	R	4.1	45	54	72	69	40	186	250	203	38	65	101	123	Heterogenous
9	R	3.3	–	–	–	–	–	–	–	–	–	–	–	–	–
10	R	6.3	33	50	54	71	35	122	112	106	31	83	133	156	Homogenous
11	L	4.8	–	–	–	–	–	–	–	–	–	–	–	–	–
12	L	10.0	32	46	71	84	38	201	161	150	33	124	127	190	Heterogenous
13	L	6.9	–	–	–	–	–	–	–	–	–	–	–	–	–
14	R	3.0	37	50	67	59	45	161	115	81	48	49	94	141	Heterogenous
15	R	11.4	38	44	49	67	29	139	217	119	28	46	100	159	Heterogenous
16	L	5.9	–	–	–	–	–	–	–	–	–	–	–	–	–
17	L	4.5	29	35	46	61	36	87	166	143	34	32	55	136	Homogenous
18	R	3.7	36	43	49	72	34	145	147	124	30	48	53	180	Homogenous
19	L	8.3	–	46	57	79	–	103	158	107	–	57	87	147	Homogenous
20	L	4.6	34	40	49	56	34	144	171	128	26	56	111	157	Homogenous
21	R	3.0	34	55	62	86	37	166	193	177	32	68	148	199	Homogenous
22	L	3.2	–	56	76	75	–	169	161	145	–	59	114	166	Homogenous

CTD, computer tomography density; HU, Hounsfield units; NC, non-contrast; .t, of tumor; CM, corticomedullary; Ne, nephrographic; Ex, excretory; .c, of normal renal cortex; .m, of normal renal medulla; R, right; L, left.

**Table 4:** CT attenuation of the tumor, renal cortex and renal medulla.

Phase	CT attenuation, HU,mean(SD,IQR)		
	Tumor	Renal cortex	Renal Medulla
Non-contrast	33.3 (6.8,31.3-36.3)	36.2 (3.9,34-37.3)	31.5 (6.7,28-33.3)
Corticomedullary	44.0 (9.1,40-50)	138.1 (34.6,124-163.5)	58.8 (22.1,45.5-65)
Nephrographic	54.4 (13.9,47.5-64.5)	155.3 (42.5,125.5-168.5)	91.9 (32.3,62-112.5)
Excretory	67.1 (11.8,60-73.5)	129.5 (30.5,110.5-144)	153.4 (23.0,137-162.5)

HU, Hounsfield units; SD, standard deviation; IQR, interquartile range.

The MR data for two cases were collected (**Table 5**). The masses showed a homogeneous and isointense signal on T1-weighted images, and the tumor signal was variable on T2-weighted images: one tumor was slightly hypointense, and the other was slightly hyperintense. After contrast administration, the tumors on T1-weighted images showed slight, homogenous and delayed enhancement. No obvious lipid content was detected in the in- and out-of-phase images of the dual chemical-shift MR sequences. High signal intensity was observed on diffusion-weighted imaging (DWI) and the apparent diffusion coefficients (ADC) of the tumors were 0.694×10^-3^ mm^2^/s and 1.115×10^-3^ mm^2^/s, respectively.

**Table 5:** MR characteristics of primary tumor.

Case No.	Side	Size (cm)	T1 signal relative to renal parenchyma	T2 signal relative to renal parenchyma	DWI	ADC(×10^-3^ mm^2^/s)	Enhancement
3	L	3.8	Isointense	Mild hypointense	Hyperintense	0.694	Mild, slow and progressive
11	L	4.8	Isointense	Mild hyperintense	Hyperintense	1.115	Mild, slow and progressive

DWI, diffusion-weighted imaging; ADC, apparent diffusion coefficient.

### Treatment and Survival Outcomes

**Table 6** summarizes the treatment and pathological outcomes of the patients. Sixteen (72.7%) patients underwent laparoscopic radical nephrectomy. Six (27.3%) patients underwent partial nephrectomy. One patient was treated *via* robot-assisted laparoscopy and the remainder *via* laparoscopy.

**Table 6:** Treatment and pathological outcomes.

Features	Value
Primary tumor therapy	
Laparoscopic partial nephrectomy, n (%)	5 (22.7%)
Robotic-assisted Laparoscopic partial nephrectomy, n (%)	1 (4.5%)
Laparoscopic radical nephrectomy, n (%)	16 (72.7%)
T Stage	
T1a, n (%)	10 (45.5%)
T1b, n (%)	8 (36.4%)
T2a, n (%)	3 (13.6%)
T2b, n (%)	1 (4.5%)
N stage	
N0, n (%)	22 (100%)
N1, n (%)	0 (0%)
M stage	
M0, n (%)	22 (100%)
M1, n (%)	0 (0%)
Tumor necrosis, n (%)	3 (13.6%)
Hemorrhage, n (%)	1 (4.5%)
Sarcomatoid change, n (%)	1 (4.5%)
Follow-up time, months, median (IQR)	59.5 (31-84)

IQR, interquartile range.

Eighteen (81.9%) patients had pT1 disease. Four (18.1%) patients had pT2 disease. Pathological examination microscopically revealed hemorrhagic, necrotic foci and sarcomatoid change in one tumor with a diameter of 8.3cm. Necrotic foci were also observed in other two tumors with the diameters of 4.5cm and 4.0cm, respectively. None of the tumors had a positive surgical margin. No masses infiltrated the perinephric and renal hilar fat, renal pelvis, calyx, or vascular. No lymph node metastasis was observed.

None of the patients received any postoperative therapy. The median follow-up duration was 59.5 months (IQR 31-84). One patient died of cerebral hemorrhage at 84 months after operation, and the others are alive without evidence of recurrence or metastasis.

## Discussion

MTSCC is a rare renal tumor that has recently been defined as a subtype of RCC (1). To date, only a limited number of MTSCC studies have been reported, and its features remain poorly defined. Histologically, MTSCC is composed of tubules lined by cuboidal and spindle cells within variable amounts of mucinous stroma (3, 4). To date, there is no description of the incidence of MTSCC in RCC in previous literature. In the present study, a total of 22 cases of MTSCC accounted for 0.52% (22/4197) of all diagnosed primary RCC cases at our institution. MTSCC has been reported to have a female predominance in previous studies (3–6). The male-to-female ratio in our series was 1:3.4, which is consistent with the results of previous studies. The median age of the cases was 52.5 years. It varies over a wide range from 32 to 66 years old. This is consistent with the majority of earlier reports, in which patients presented at a wide age range from 17 to 86 years old (3–6). Similar to other renal masses, MTSCC is often asymptomatic and occasionally presents with symptoms such as flank pain or hematuria (4, 5). In our study, only two patients presented with symptom of flank pain. Their tumors were with larger diameters of 9.0cm and 11.4cm respectively.

Given the rarity of MTSCC, there are few data that contribute to defining its imaging features. In this study, the ultrasound, CT and MRI imaging findings of MTSCC were evaluated. To our knowledge, this is the largest single-center study to analyze the multimodal imaging features of MTSCC.

The description of ultrasound imaging of MTSCC has been very rare in prior studies. Sahni et al. reported ultrasonographic features of two cases of MTSCC. The masses were well-marginated and homogenously hypoechoic (7). Yan et al. also described ultrasound appearance in two cases (8). The lesion was well-defined, homogenous, and slightly hypoechoic in case 1, and mildly heterogeneous and slightly hyperechoic in case 2. Zhang et al. reported ultrasound imaging in 6 cases and all masses were predominantly hypoechoic (9). Similar to previous reports, most cases in our study had well-marginated, homogenously hypoechoic lesions. However, heterogeneous or hyperechoic masses can still be observed in a few cases. Color Doppler ultrasound revealed peripheral blood flow signals and no obvious blood flow into the masses. CEUS showed slow in, fast or simultaneous out of the contrast agent and slightly heterogeneous enhancement, which indicated homogenous hypovascular features of the masses.

We further focused on the CT imaging features of MTSCC. Non-contrast CT scan showed isodense lesions compared to the normal renal parenchyma in the present study. The enhancement of these tumors was progressive, slow, and substantially less than that of the corresponding cortex in all enhanced phases. The maximum enhancement was observed in the latest phase (nephrographic or later). The homogenicity of enhancement is one of the most important features of CT for the diagnosis of tumors. Cornelis et al. reported that the enhancement of MTSCC tumors was heterogeneous (10). The tumor size ranged from 6 to 110mm. Kenney et al. reported that small MTSCC tumors show homogenous enhancement (11). Heterogenous enhancement was observed mostly in tumors >5cm in size (11). In our study, enhancement was homogenous in most cases, even in large tumors.

All tumors in our series were well-marginated. This is consistent with the results of most previous studies (10, 11). Zhu et al. reported 14 cases of MTSCC with poorly defined margins on the delayed phase of contrast-enhanced CT (12). In our study, most of the lesions were spherical or ovoid. Lobulated contours, calcification, necrosis or cystic components have also been observed in a few cases. No hemorrhage was found even in large tumors>10cm. Kenney et al. reported the appearance of intralesional hemorrhage on non-contrast CT in two cases (11). The diameters of the tumors were 14cm and 5 cm, respectively. No fatty content was found in our cases, which is similar to previous reports (10, 11).

MRI is generally superior to ultrasound and CT in the diagnosis of RCC and the differentiation of its subtypes (13). However, reports concerning the MR imaging features of MTSCC are much fewer than those of CT. Sahni et al. reported that the masses of MTSCC presented with isointense signals on T1-weighted images and hypo-, iso-, or hyperintense signals on T2-weighted images (7). Cornelis et al. reported high or heterogeneous signal intensity on T1-weighted images and high, low, or heterogeneous signal intensity on T2-weighted images (10). Enhancement of the masses was heterogeneous, slow and progressive. In our study, the masses were isointense with the normal renal cortex on T1-weighted images. On T2-weighted images, one tumor showed a slightly lower signal, and the other showed a slightly higher signal. High signal intensity was observed on DWI. After contrast administration, the tumors on T1-weighted images showed slight, homogenous and delayed enhancement, which is similar to the features of enhancement on CT. No lipid content was observed. Based on the limited findings above, it seems that MTSCC may have a variable appearance on MRI. Cornelis et al. speculated that these variations may be explained by the different proportions of components of the tumor itself, which are composed of cells set within mucinous or myxoid stroma (10). However, in our study, there were no obvious differences in the proportions of histological components between tumors with high or low T2-weighted signal intensity. Therefore, further investigation is required for a rational explanation.

The assessment of tumor vascularity is particularly important for tumor characterization (14). Based on the imaging findings, MTSCC showed a common imaging appearance, indicating that it was a hypovascular renal tumor. Enhancement is the most valuable parameter for the differentiation of RCC subtypes (14–16). MTSCC is almost universally slightly and homogeneously enhanced on different imaging examinations. Maximum enhancement of MTSCC on CT imaging appears in the nephrographic or excretory phase. These features differ from those of clear cell RCC, the enhancement of which is usually heterogeneous and greater than that of the renal cortex. There are overlapping imaging features between MTSCC and other hypovascular RCC subtypes such as papillary RCC, chromophobe RCC and collecting duct carcinoma. Therefore, it remains challenging to differentiate MTSCC from other hypovascular RCCs using only imaging techniques.

Owing to the paucity of the disease, there are no sufficient data to guide therapy for MTSCC currently. In our series, imaging features were the determining factor for choosing candidate treatment modalities. Patients with localized lesion were treated with partial or radical nephrectomy in laparoscopic or robot-assisted manner. The survival outcomes were favorable. No aggressive behavior, such as recurrence or metastasis, was observed in any of the cases. Recurrence and metastasis rarely occur after surgical resection of tumors in previous reports (17–20). MTSCCs are generally low malignant. Therefore, even for cases with large tumors, nephron-sparing surgery should be considered as an alternative. This will be helpful in preserving postoperative renal function of the patients.

At the beginning of our study, we have excluded two cases whose diagnoses were revised after further pathological re-review. Their prognoses were poor. Case 1 is a 70-year-old man presented with a 1-month history of high fever. 18F-FDG-PET/CT scan showed a 2-cm right renal mass, a 2-cm left adrenal mass, a right renal hilar lymph node metastasis and widespread bone metastases. The patient underwent a laparoscopic radical nephrectomy and retroperitoneal lymphadenectomy. Microscopically, renal capsule, hilar lymph node and ipsilateral adrenal invasion were observed. The sunitinib has been administrated after surgery for only one month because an adverse effect, renal impairment, developed. The patient died of multiple organ failure due to the progression of the disease 3 months after the nephrectomy. Case 2 is a 77-year-old man presented with a 5-month history of flank pain and microscopical hematuria. CT scan revealed a 3.1-cm left renal mass. Accordingly, laparoscopic radical nephrectomy was performed. Renal pelvic invasion was found microscopically. Follow-up imaging examination revealed multiple metastatic lesions. The patient finally died due to the disease 7 months after surgery. Although aggressive behavior has been reported in a few cases (17–20), MTSCC is generally an indolent RCC subtype. Accordingly, clinicians should be cautious before diagnosing MTSCC in patients with infiltrating or metastatic lesions.

## Conclusions

In summary, MTSCC presents with a female predilection and is universally indolent. It should be deliberate for a pathologist to diagnose MTSCC in renal tumors with aggressive behavior. The treatment outcomes for patients with localized MTSCC are excellent. Preoperative diagnosis is meaningful and helpful in making the decision to preserve the kidney. Multimodal imaging demonstrated that MTSCC is a hypovascular renal tumor that is significantly different from clear cell RCC. However, its imaging features overlap with those of other hypovascular renal tumors. Further studies are needed to fully characterize the features of this rare RCC variant.

## Data Availability Statement

The original contributions presented in the study are included in the article/supplementary material. Further inquiries can be directed to the corresponding authors.

## Ethics Statement

The studies involving human participants were reviewed and approved by Institutional Review Board of Jinling Hospital. Written informed consent for participation was not required for this study in accordance with the national legislation and the institutional requirements.

## Author Contributions

JG and WC had full access to all the data in the study and takes responsibility for the integrity of the data and the accuracy of the data analysis. Study conception and design: JG and WC. Data acquisition: XX, JZ, ZW, QX, and PH. Data analysis and interpretation: XX, JZ, XZ, and CT. Drafting the manuscript: XX and XZ. Critical revision of the manuscript for scientific and factual content: XX and XZ. Statistical analysis: CS and JD. Obtaining funding: XX. Supervision: JG and WC. All authors contributed to the article and approved the submitted version.

## Funding

This work was supported by National Natural Science Foundation of China (NSFC81972841).

## Conflict of Interest

The authors declare that the research was conducted in the absence of any commercial or financial relationships that could be construed as a potential conflict of interest.

## Publisher’s Note

All claims expressed in this article are solely those of the authors and do not necessarily represent those of their affiliated organizations, or those of the publisher, the editors and the reviewers. Any product that may be evaluated in this article, or claim that may be made by its manufacturer, is not guaranteed or endorsed by the publisher.

## Abbreviations

ADC, apparent diffusion coefficient; AJCC, American Joint Committee on Cancer; CEUS, contrast-enhanced ultrasonography; CM, corticomedullary; DWI, diffusion-weighted imaging; Ex, excretory; MTSCC, mucinous tubular and spindle cell carcinoma; NC, non-contrast; Ne, nephrographic; PACS, picture archiving and communication system; RCC, renal cell carcinoma; ROI, region of interest; TNM, tumor node and metastasis.
